# Specific residues modulate the antiviral activity of the extracellular vesicle-derived effector SH3b domain from *Lactobacillus johnsonii* N6.2

**DOI:** 10.3389/fimmu.2026.1977036

**Published:** 2026-10-22

**Authors:** Asra B. Sharjeel, Javier Rosero, Chantal Ronacher, Melissa K. Jones, Claudio F. Gonzalez, Graciela L. Lorca

**Affiliations:** Department of Microbiology and Cell Science, Genetics Institute, Institute of Food and Agricultural Sciences, University of Florida, Gainesville, FL, United States

**Keywords:** bacterial extracellular vesicles, bacterial Src homology 3 domain (SH3b), interferon, *Lactobacillus johnsonii*, macrophages, murine norovirus MNV-1, NF-ĸB, probiotics

## Abstract

*Lactobacillus johnsonii* N6.2-derived extracellular vesicles (EVs) provide protection against murine norovirus (MNV-1) both *in vitro* and *in vivo*. Within the proteins enriched in EVs, the SH3b2 domain of Sdp was shown to be sufficient to recapitulate the antiviral effects shown by EVs. In this report, we compared the effects of six SH3b domains of Sdp and identified amino acids expected to be important for interactions with host proteins. We found that SH3b2 showed the highest activity in inducing NF-ĸB/AP-1 and IRF pathways in RAW macrophage reporter cell lines. Structure-guided site-directed mutagenesis of SH3b2 revealed residues in SH3b2 potentially responsible for interactions with host proteins. SH3b2-G501D had significantly lower stimulation of NF-ĸB/AP-1 and IRF pathways. In addition, SH3b2-G501D showed reduced *IL10* and *Oas1* mRNA levels, resulting in an impaired MNV-1 antiviral response in RAW 264.7 cells. mCherry fusions to SH3b2-G501D confirmed that the effects observed were not related to reduced uptake into macrophages, as quantified by confocal microscopy. Additionally, the role of the C-terminal linker sequence in Sh3b2, which is absent in SH3b6, in uptake or signaling in macrophages was evaluated. It was found that deletion of the C-terminal linker (SH3b2ΔC2 treatment) resulted in reduced promoter activity, while SH3b6::C2 partially regained function through restoration of uptake in macrophages. These results indicate that the C-terminal linker plays an important role in internalization by macrophages. We propose that once internalized, SH3b2 mimics eukaryotic SH3 domains involved in signal transduction to activate the cellular innate immune response in macrophages.

## 1 Introduction

Once considered cellular byproducts, extracellular vesicles (EVs) are now recognized as key regulators of cellular communication and carriers of biologically active molecules. EVs are produced by all domains of life, and their functions vary depending on the characteristics of the organism from which they originate (1–5). These lipid bilayer-enclosed particles, ranging in size from approximately 20 to 500 nm in diameter, are secreted by both Gram-negative and Gram-positive bacteria as well as eukaryotic cells (6, 7). They take part in cellular communication and cargo delivery among cells, and carry a wide range of proteins, lipids, polysaccharides, and nucleic acids (8, 9). EVs from pathogenic microorganisms may transport toxic cargo to the host cells. For example, EVs produced by *Staphylococcus aureus* induced cytotoxicity in human A549 lung epithelial cells and caused hemolysis in rabbit-derived erythrocytes (8, 10). In contrast, EVs derived from beneficial microorganisms such as commensals or probiotics are shown to have anti-inflammatory effects. For example, *Bifidobacterium longum* EVs were shown to induce the production of IL-10 in mouse splenocytes (11, 12). Probiotics-derived EVs demonstrate diverse health-related benefits that include improved overall gut health, immune modulation, neurological effects, and infection control (3, 13). *Lactiplantibacillus plantarum* and *B. longum* EVs activate macrophages (Mp) via toll-like receptor 2 (TLR2) (14) while *Latilactobacillus sakei* induces the production of IgA in Peyer’s patch cells to control infection (15).

Probiotics-derived EVs can carry beneficial effector molecules that can be delivered to host cells. We previously reported that a probiotic bacterial strain, *Lactobacillus johnsonii* N6.2, produces EVs (8, 16–18). Our earlier work demonstrated that EVs purified from *L. johnsonii* N6.2 (EVs) are internalized by eukaryotic cells via clathrin- and dynamin-mediated endocytosis, eliciting immunomodulatory effects in the host’s intestinal tract (8, 19). We also found that treatment of THP-1 Mp with EVs resulted in Mp polarization toward an M2b tolerogenic phenotype. The phenotype was confirmed by the significantly increased expression of biomarkers like TNF-α, IL-1β, and IL-10. In Mp, EVs stimulate the phosphorylation of Y705 in signal transducer and activator of transcription 3 (STAT3), which indirectly promotes IL-10 expression (20). Additionally, EVs elicited the stimulation of the innate antiviral response through increased expression of the 2′–5′–oligoadenylate synthetase (OAS) pathway in both RAW 264.7 murine and THP-1 human Mp. The stimulation of antiviral responses positively correlated with decreased murine norovirus (MNV-1) titers *in vitro* and in C57BL/6 mice (21).

To identify effector molecules enriched in EVs responsible for their immunomodulatory effects, omics analysis revealed the presence of a distinct lipid, RNA, and protein profile of EVs comparing to the cell membrane or whole bacterial cell (8, 19). Among the proteins enriched on EVs, SH3b2 and SH3b6 domains of Sdp (SH3 domain-containing protein) were initially selected as biomarkers of EVs (8). Both domains were detectable by IgA and IgG anti-commensal antibodies (ACab) in the plasma samples obtained from subjects in a clinical trial who received *L. johnsonii* N6.2 for 8 weeks (8, 22).

Sdp is a large membrane-bound protein with a predicted molecular weight of 102 kDa. It has an N-terminal glycosyl hydrolase family 25 (GH25) domain followed by six tandem repeats of SH3b domains, connected by variable linker sequences (8, 21). The Src homology 3 (SH3) domain family is widespread among organisms and has been described as having modular binding functions (23–25). In eukaryotes, these β barrel fold domains consist of approximately 60 amino acids with high sequence identity. SH3 domains are predominantly associated with signaling proteins like hematopoietic progenitor kinase-1 interacting protein (HIP 55), growth factor receptor bound protein 2 (Grb2), or CT10 regulator of kinase (CRK) (23, 26). SH3 domains typically interact with proline-rich motifs or PxxP motifs on their targets (24, 25, 27–30). Such interactions allow SH3 domains to assemble multiprotein complexes with important roles that include endocytosis, modification of the actin cytoskeleton, apoptosis, or cell proliferation. Disruption of SH3-mediated signal transduction can lead to immune system-related disease progression, neurodegenerative diseases and/or cancer (23, 24, 26, 31). In bacterial cells, SH3 domains are called bacterial SRC homology 3 (SH3b) domains that are homologs of eukaryotic SH3 domains (32, 33). They are widely found among bacterial and bacteriophage proteins. SH3b domains have been found associated with proteins that interact with bacterial cell wall motifs like the peptidoglycan pentaglycine bridge (34, 35). Among the best characterized examples are SH3b domains involved in enzymatic activity. For instance, the SH3b domain of lysostaphin contributes to its bacteriolytic function by facilitating the cleavage of the bond between the third and fourth glycine residues in the pentaglycine bridge of the peptidoglycan in *Staphylococcus* species (36, 37).

SH3b domains are also widely found in lactic acid bacteria (8, 38). Interestingly, while most SH3b domain-containing proteins have one or two SH3b domains, very few species have four to six SH3b domains (8). Previously, we evaluated whether two purified SH3b domains would stimulate similar responses in Mp cell lines. We reported that SH3b domain 2 (SH3b2) and 6 (SH3b6) of Sdp are not equal at inducing host immune responses. It was shown that SH3b2 domain can induce the expression of nuclear factor kappa B, (NF-ĸB) and interferon regulatory factor (IRF) reporter genes in RAW-DUAL™ KO-TLR4 murine Mp while SH3b6 exhibited very low activity. Furthermore, *in vivo*, SH3b2 showed a similar effect as EVs in significantly reducing MNV-1 viral load in the distal ileum of C57BL/6 mice (21).

In this report, we aim to identify key residues involved in the activity of SH3b2 domain of Sdp. We hypothesize that SH3b domains of Sdp are effectors involved in uptake and trafficking of EVs. Once internalized, we propose that the SH3b domains modulate cellular immune function through protein-protein interactions with eukaryotic proteins by molecular mimicry. Here, structural, structural modeling followed by site-directed mutagenesis was utilized to identify residues in SH3b2 predicted to interact with proline-rich domains of eukaryotic proteins. Using this approach, we identified residues that can increase or inactivate signal transduction, immune stimulation, and the antiviral activity of SH3b2. In addition, we uncovered the significant contribution of the inter-SH3b-linker sequences on the uptake of SH3b2 into eukaryotic cells.

## 2 Materials and methods

### 2.1 Bioinformatics and Structural model analysis

Multiple sequence alignments (MSA) of SH3b2 and eukaryotic SH3 domains were conducted using MultAlin and Jalview tools (39, 40). Structural modeling of SH3b2 and SH3b6 domains was performed via the Phyre2.2 server using standard settings (41). To evaluate structural similarity to eukaryotic SH3 domains, the predicted structure of SH3b2 was aligned with a representative eukaryotic SH3 domains Cortactin (PDB: 2D1X, (42)) and Grb2 (PDB: 1AZE, (43)) using UCSF ChimeraX 1.9 structural alignment matchmaker tool with pairwise alignment settings (42–44). The structural alignment was further edited and analyzed using UCSF ChimeraX 1.9. Secondary structure and loop architecture were annotated using the DSSP implementation in UCSF ChimeraX 1.9, enabling the identification of β-strands and the presence of loops in SH3b2 and SH3b6.

### 2.2 Cloning and site-directed mutagenesis

Domains SH3b1 (amino acids 310-427), SH3b3 (534–639), SH3b4 (640–745) & SH3b5 (746–849) of Sdp were cloned into the p15TV-L vector using In-Fusion^®^ Snap Assembly EcoDry™ Master Mix (Takara Bio USA, Inc.). Primers were designed to amplify the domains from genomic DNA extracted from *L. johnsonii* N6.2 as described earlier (8) (**Table 1**). Purified PCR fragments were cloned into the linearized p15TV-L plasmid, transformed into *E. coli* DH5α competent cells and plated on LB medium with ampicillin (100 µg/ml). Recombinant plasmids were confirmed via sequencing using T7 FWD and RV primers. For protein purification, recombinant plasmids were transformed into ClearColi BL21 (DE3) cells (LGC, Biosearch Technologies, CA, USA). SH3b2 (428–533) and SH3b6 (850–935) cloned into the p15TV-L vector were available from the previous work (8, 21).

**Table 1:** Primer sequences used in this study.

Locus tag	Primer name	Primer sequence (5’-3’)	Source
Primers for cloning			
T285_RS00825	SH3b1 FWD	TTGTATTTCCAGGGCCCAAACACAACCACTAATGTAAA	(8)
T285_RS00825	SH3b1 RV	CAAGCTTCGTCATCACTTTTCTACTGGCTT	*
T285_RS00825	SH3b2 FWD	TTGTATTTCCAGGGCCCAAGTACAAACACTAACGTAAATA	(8)
T285_RS00825	SH3b2 RV	CAAGCTTCGTCATCACTTTTCTGCTGGTTGACTT	(8)
T285_RS00825	SH3b3 FWD	TTGTATTTCCAGGGCCCAAACATAACCACTAATGTAAACAGT	*
T285_RS00825	SH3b3 RV	CAAGCTTCGTCATCATTACTTTTCTGCTGGTTGACTTGGTTT	*
T285_RS00825	SH3b4 FWD	TTGTATTTCCAGGGCCCAAACATAACCACTAATGTAAACAGT	*
T285_RS00825	SH3b4 RV	CAAGCTTCGTCATCACTTTTCTGTTGGTTGACTTGGTTTGTT	*
T285_RS00825	SH3b5 FWD	TTGTATTTCCAGGGCCCAAACATAACCACTAACGTAAAC	*
T285_RS00825	SH3b5 RV	CAAGCTTCGTCATCAAGTAGTTTGTCGAACAGGCTTATTAGCTACTGCATT	*
T285_RS00825	SH3b6 FWD	TTGTATTTCCAGGGCCCTAAGCAAGTAACTACTAATAAC	(8)
T285_RS00825	SH3b6 RV	CAAGCTTCGTCATCATCTGTAAGTTCCCCATGC	(8)
T285_RS00825	SH3b2ΔC2 FWD	TTGTATTTCCAGGGCCCAAGTACAAACACTAACGTAAATA	*
T285_RS00825	SH3b2ΔC2 RV	CAAGCTTCGTCATCAACGGTATGTTCCCCATGCTTGGCCATTATCACGTCCTAC	*
T285_RS00825	SH3b6::C2 (1) FWD	TTGTATTTCCAGGGCCCTAAGCAAGTAACTACTAATAAC	*
T285_RS00825	SH3b6::C2 (1) RV	CAAGCTTCGTCATCAAGTTGCTGCAGAACCTTCCTTGAATCCCCATGC	*
T285_RS00825	SH3b6::C2 (2) FWD	TTGTATTTCCAGGGCCCTAAGCAAGTAACTACTAATAAC	*
T285_RS00825	SH3b6::C2 (2) RV	CAAGCTTCGTCATCATTTGTTAGTAGTTTGAGCTGGCTTAGTTGCTGC	*
T285_RS00825	SH3b6::C2 (3) FWD	TTGTATTTCCAGGGCCCTAAGCAAGTAACTACTAATAAC	*
T285_RS00825	SH3b6::C2 (3) RV	CAAGCTTCGTCATCACTTTTCTGCTGGTTGACTTGGTTTGTTAGTAGT	*
mCherry	mCherry FWD	TTGTATTTCCAGGGCATGGCCATCATCAAGGAGTTCATG	*
mCherry	mCherry RV	CAAGCTTCGTCATCAGTTTCCGGACTTGTACAGCTCGTCCAT	*
T285_RS00825	mCh-SH3b2 FWD	ATGGACGAGCTGTACAAGTCCGGAAACCCAAGTACAAACACT	*
T285_RS00825	mCh-SH3b2 RV	AGTGCGGCCGCAAGCTTCGTCATCACTTTTCTGCTGGTTGACT	*
T285_RS00825	mCh-SH3b6 FWD	ATGGACGAGCTGTACAAGTCCGGAAACCCTAAGCAAGTA	*
T285_RS00825	mCh-SH3b6 RV	AGTGCGGCCGCAAGCTTCGTCATCATCTGTAAGTTCCCCATGC	*
T285_RS00825	mCh-SH3b2ΔC2 FWD	ATGGACGAGCTGTACAAGTCCGGAAACCCAAGTACAAACACT	*
T285_RS00825	mCh-SH3b2ΔC2 RV	AGTGCGGCCGCAAGCTTCGTCATCATCTGTAAGTTCCCCATGC	*
T285_RS00825	mCh-SH3b6::C2 FWD	ATGGACGAGCTGTACAAGTCCGGAAACCCTAAGCAAGTA	*
T285_RS00825	mCh-SH3b6::C2 RV	AGTGCGGCCGCAAGCTTCGTCATCACTTTTCTGCTGGTTGACT	*
Primers for mutagenesis			
T285_RS00825	SH3b2_N470A FWD	GCTTCCAACAGCGACTGAAGTTAAGTAC	*
T285_RS00825	SH3b2_N470A RV	ATTGCAATTACTTTACTGTTAG	*
T285_RS00825	SH3b2_E472A FWD	AACAAATACTGCGGTTAAGTACGATGC	*
T285_RS00825	SH3b2_E472A RV	GGAAGCATTGCAATTAC	*
T285_RS00825	SH3b2_E472V FWD	GGAAGCATTGCAATTAC	*
T285_RS00825	SH3b2_E472V RV	GCATCGTACTTAACCACAGTATTTGTT	*
T285_RS00825	SH3b2_G501A FWD	TGATAATGGCGCGGCATGGGGAAC	*
T285_RS00825	SH3b2_G501A RV	CGTCCTACTAAGTAACC	*
T285_RS00825	SH3b2_G501D FWD	TTACTTAGTAGATCGTGATAATGGC	*
T285_RS00825	SH3b2_G501D RV	CCATATTCATTATTTGCACG	*
T285_RS00825	SH3b2_D503A FWD	AGTAGGACGTGATAATGGCCAAG	*
T285_RS00825	SH3b2_D503A RV	AAGTAACCATATTCATTATTTGC	*
T285_RS00825	SH3b2_Q506A FWD	TGATAATGGCGCGGCATGGGGAAC	*
T285_RS00825	SH3b2_Q506A RV	CGTCCTACTAAGTAACC	*
T285_RS00825	SH3b2_W508A FWD	TGGCCAAGCAGCGGGAACATTCAAG	*
T285_RS00825	SH3b2_W508A RV	TTATCACGTCCTACTAAGTAAC	*
T285_RS00825	SH3b2_T510A FWD	AGCATGGGGAGCGTTCAAGGAAG	*
T285_RS00825	SH3b2_T510A RV	TGGCCATTATCACGTC	*
Primers for qRT-PCR			
*Gapdh*	murine Gapdh FWD	AGTATGACTCCACTCACGGCAAAT	(21)
*Gapdh*	murine Gapdh RV	GTCTCGCTCCTGGAAGATGGT	(21)
*Oas1b*	murine Oas1b FWD	TGGACCTAGGATGGAGCAGG	(21)
*Oas1b*	murine Oas1b RV	GCACACAGGGCATCTATGACT	(21)
*Il10*	murine Il10 FWD	TGGGTTGCCAAGCCTTATCG	(21)
*Il10*	murine Il10 RV	CAGCTTCTCACCCAGGGAAT	(21)
*MNV1*	MNV-1 FWD	GTGCGCAACACAGAGAAACG	(21)
*MNV1*	MNV-1 RV	CGGGCTGAGCTTCCTGC	(21)

*Original to this work.

Variants of the SH3b2 domain of Sdp were created by single-residue point mutations. Primers for site-directed mutagenesis were designed using the online software NEB Base Changer v2.8.4 (New England Biolabs, NEB, Ipswich, MA, USA) (**Table 1**). SH3b2 variants N470A, E472A, E472V, G501A, G501D, D503A, Q506A, W508A, T510A were created via PCR-based site-directed mutagenesis using Q5^®^ Site-Directed Mutagenesis Kit (NEB). Plasmid vector p15TV-L_SH3b2 was utilized as a template, and successful mutations were confirmed by Sanger sequencing. Mutant SH3b2 proteins were overexpressed in ClearColi BL21 (DE3) (LGC, Biosearch Technologies, CA, USA) and purified as previously described (8).

SH3b2ΔC2 protein was constructed by deleting the C-terminal sequence (TFKEGSAATKPAQTTNKPSQPAEK) from the SH3b2 domain. Primers were designed to perform the sequence deletion during cloning (**Table 1**). The Sh3b2 domain was amplified via PCR using genomic DNA of *L. johnsonii* N6.2 as a template. The amplified sequence was cloned into the p15TV-L plasmid vector as described earlier.

SH3b6::C2 recombinant protein was constructed by adding the SH3b2 C-terminal (TFKEGSAATKPAQTTNKPSQPAEK) into SH3b6. Cloning into p15TV-L plasmid vector was performed in three steps by using specific sets of primers to add the insertion sequence (**Table 1**). In the first step, TFKEGSAAT residues were added to SH3b6 (SH3b6::C2 Set 1). The second cloning step added KPAQTTNK residues to construct SH3b6::C2-Set 2, using SH3b6::C2 Set 1 as a template. The third cloning step added PSQPAEK residues to SH3b6::C2-Set 2 to obtain the final construct, SH3b6::C2. The insertions were confirmed by sequencing after each cloning step. Recombinant proteins were overexpressed in ClearColi BL21 (DE3) (LGC, Biosearch Technologies, CA, USA) and were purified as described above.

### 2.3 Protein purification

Cells were grown in LB media with 100 μg/mL ampicillin at 37 °C to an OD600 = 0.7. Gene overexpression was achieved using 0.5 mM isopropyl β-d-1-thiogalactopyranoside (IPTG) followed by incubation at 16 °C for 16 h. The cell pellet was collected by centrifugation at 15000 x *g* for 25 min and was frozen at -80 °C until purification. Pellets were resuspended in binding buffer containing 5% glycerol, 50 mM Tris pH 8, 500 mM NaCl, and 5 mM imidazole, followed by lysis using a french press. The ruptured cells were centrifuged at 35,000 x *g* for 45 min to remove cell debris, and the cell-free extract was applied to a Ni^2+^ affinity column. The column was washed twice with wash buffer (5% glycerol, 50 mM Tris pH 8, 500 mM NaCl, and 25 mM imidazole). Proteins were eluted with elution buffer (5% glycerol, 50 mM Tris pH 8, 500 mM NaCl and 250 mM imidazole). The obtained proteins were dialyzed overnight in dialysis buffer (10 mM Tris pH 8, 2.5% glycerol, 500 mM NaCl). Protein aliquots were stored at -80. Bradford assay (Bio-Rad, Hercules, CA, USA) was used to measure protein concentrations using bovine serum albumin (BSA; Gold BioTechnology, St. Louis, MO, USA) as a standard.

### 2.4 Differential scanning fluorimetry

DSF was performed by mixing 5x SYPRO orange dye (Thermo Fisher, MA, USA) with 35 uM of SH3b2, SH3b6, G501D, W508A, SH3b2ΔC2, SH3b6::C2 in a 384-well plate. Dialysis buffer with dye was utilized as a negative control. Fluorescence was measured in a QuantStudio 6 Flex machine (Thermo Scientific, Waltham, MA, USA) when the temperature rose from 25 to 95˚C, at the rate of 1˚C/min. Boltzmann sigmoidal equation, F (T) = F (min) + [F (max) - F (min)/[1 + exp ((Tm - T/a)] where F (min) and F (max) are the minimum and maximum fluorescence readings, was used to fit the transition region fluorescence data to determine the melting temperatures (Tm). Curve fitting and statistical analysis was performed using GraphPad Prism 9.491 software (GraphPad Software, La Jolla, CA, USA).

### 2.5 *In vitro* cell reporter assays

RAW-Lucia™ ISG Cells, RAW-Blue™ Cells and RAW DUAL™ KO-TLR4 Mp (Invivogen, San Diego, CA, USA) were seeded in a 96-well plate at 2.00E + 04 cells/well in complete Dulbecco’s Modified Eagle Medium (DMEM) containing 1% Penicillin/Streptomycin (Pen/Strep), 10% Fetal Bovine Serum (FBS) (Gibco GlutaMAX, Thermo Scientific, USA). Cells were allowed to reach confluency at 37 °C with 5% CO_2_. Where indicated, cells were treated with purified protein domains (SH3b1, SH3b2, SH3b3, SH3b4, SH3b5, and SH3b6) or SH3b2 mutant proteins at 1.5 μg/mL in triplicate. After 18 h of incubation at 37 °C with 5% CO_2_, the supernatants were collected, and cellular debris was separated by centrifugation at 13000 rpm for 3 minutes. Cell-free supernatants were used for enzymatic determinations of secreted embryonic alkaline phosphatase activity (SEAP) and Lucia Luciferase (Luc) using Quanti-Blue and Quanti-Luc reagents, respectively (Invivogen San Diego, CA, USA). Macrophage inflammatory protein-2 (MIP-2) promoter fusion to SEAP quantifies NF-ĸB/activator protein 1 (AP-1) signaling pathways in RAW-Blue™ Cells and NF-ĸB signaling in RAW DUAL™ KO-TLR4 Mp. IFN-stimulated response elements (ISRE) promoter fusion to the Lucia Luciferase gene quantifies the activation of Interferon signaling pathways (IRF3/IRF9) in RAW-Lucia™ ISG Cells and RAW DUAL™ KO-TLR4 Mp.

For the measurement of SEAP activity, cell supernatants were mixed with Quanti-Blue solution (Invivogen, San Diego, CA, USA) and incubated for 4 hours for KO TLR4 and for 2 hours for RAW Blue in a plate reader in a plate reader at 37 °C. The plates were read continuously (every 5 min). The enzymatic activity was expressed as arbitrary units (AU), calculated by inspecting the linear portion of the slope to select changes in optical density (OD) over time as [OD(t1)-OD(t2)]/time interval (in minutes).

Luciferase activity was quantified using Quanti-Luc solution (Invivogen San Diego, CA, USA) via luminescence using an automatic injector of a CLARIOstar microplate reader (BMG Labtech, Cary, NC, USA). Relative light units (RLU) obtained from the Lucia Luciferase assay were calculated as percentages after being normalized to the negative control.

2′3′-cyclic guanosine monophosphate-adenosine monophosphate (2’ 3’-cGAMP) (5 μg/mL) was used as a negative control for measuring the expression of the MIP-2 promoter, whereas 2’ 3’-cGAMP (5 µg/mL) was a positive control for the measurement of ISRE promoter activity. Mock was utilized as a negative control for both assays. Lipopolysaccharide (LPS) (1 μg/mL) was used as a negative control for RAW DUAL™ KO-TLR4 Mp and as a positive control for WT cell lines.

### 2.6 Murine Norovirus (MNV-1) production

MNV-1 was produced using HEK 293T cells transfected with 5 mg of pSPMNV-1.CW3 plasmid to express recombinant MNV-1 genome. Next, RAW264.7 cells were infected by supernatants collected from the transfected HEK 293T cells (MOI = 0.05). RAW 264.7 cells were evaluated for ~90% cytopathic effect (CPE) 36–48 hours post infection (hpi). To obtain purified viral pellets, RAW264.7 cell supernatant containing viral particles was collected followed by ultracentrifugation through a 25% sucrose cushion. Viral pellets were resuspended in PBS titrated using 50% tissue culture infectious dose (TCID_50_) assay as previously reported (45–47). TCID_50_/ml was determined by the Reed-Muench method. A mock inoculum was prepared in parallel using HEK 293T cells transfected with empty vector. Virus stocks were stored at -80 ˚C and were pre-incubated in media at 37 ˚C for 1h with agitation prior to infection.

### 2.7 MNV-1 infection

RAW 264.7 Mp were plated 5.00E + 05 cells/mL in a 6-well plate and grown for 48 h to reach confluence (~1.00E+06 cells). Mp were treated with 1.5 μg/mL of SH3b2, G501A, G501D, D503A, W508A, or controls using fresh DMEM media for 6 h. After treatment, media with the proteins and controls were removed and Mp were infected with Murine Norovirus inoculum (MNV-1). Multiplicity of infection (MOI = 5) was calculated from TCID_50_ titers. MNV-1 inoculum was prepared by incubating MNV-1 (MOI = 5) in 500uL of DMEM at 37 °C for 1 h with gentle agitation prior to infection. After infection, media containing viral particles was removed, and cells were washed with PBS twice before incubation with fresh media for 6 h or 18 h followed by RNA extraction and qRT-PCR as described earlier (21).

### 2.8 mRNA extraction and qRT-PCR

RNA was extracted from the cells using Qiagen RNeasy Miniprep (QIAGEN, Germantown, MD, USA). DNase Turbo kit (Thermo Scientific, Waltham, MA, USA) was used to treat purified RNA. cDNA was synthesized from purified RNA using iScript™ cDNA Synthesis Kit (Bio-Rad, Hercules, CA, USA). qRT-PCR was performed using QuantStudio 6 Flex (Thermo Scientific, Waltham, MA, USA). SYBR Green Master Mix (Applied Biosystems, Foster City, CA, USA) was used for qRT-PCR, using glyceraldehyde 3-phosphate dehydrogenase (*GAPDH*) as housekeeping gene. The gene expression changes (C_t_ values) between genes were compared to the housekeeping gene using 2^-ΔΔ^*^Ct^*method. (qRT-PCR primer sequences are listed in **Table 1**). Data were analyzed and visualized using GraphPad Prism 9.491 software (GraphPad Software, La Jolla, CA, USA). Statistical tests were performed using one-way ANOVA and a *post hoc* Tukey test using three biological and two technical replicates.

### 2.9 Construction of mCherry-Sh3b protein fusion

mCherry was fused to the different SH3b domains and mutant versions of SH3b2 (mCh-SH3b2, mCh-SH3b6, mCh-SH3b2ΔC2, mCh-SH3b6::C2, mCh-E472A, mCh-E472V, mCh-G501A, mCh-G501D, mCh-D503A, mCh-W508A) to visualize protein trafficking using confocal microscopy. First, mCherry was amplified by PCR from pmR-mCherry (Takara Bio USA, Inc.) and cloned into p15TV-L as mentioned previously. Recombinant plasmids were used to clone the SH3b domains downstream. To that end, p15-mCherry was linearized using BsgRI and NotI (NEB). Primers used are described in **Table 1**. Cloning was performed using In-Fusion^®^ Snap Assembly EcoDry™ Master Mix (Takara Bio USA, Inc.) as per manufacturers guidelines. Successful recombinants were confirmed using Sanger sequencing with T7 promoter primers followed by transformation into ClearColi BL21 (DE3) (LGC, Biosearch Technologies, CA, USA). Fusion proteins were purified as described earlier using 1X Halt™ Protease Inhibitor Cocktail, EDTA-Free (100X) in all the buffers.

### 2.10 Confocal microscopy

THP-1 macrophages were propagated in Roswell Park Memorial Institute (RPMI 1640) media with 10% FBS and 1% Pen/Strep. Cells were seeded and activated with 100 nM/mL of Phorbol 12-myristate 13-acetate (PMA) in a 6-well plate containing German round coverslips (Electron Microscopy Sciences, Fisher Scientific, USA) at 1.00E + 6 cells/well for 24 h at 37 ˚C and 5% CO_2_. Media with PMA was removed, and cells were incubated in prewarmed fresh media for two hours before treatment. After incubation, cells were kept either untreated as a negative control or treated with 5 μg/mL purified proteins for 1 h at 37 °C and 5% CO_2_. Proteins evaluated were: mCherry (mCh) (control), or fusion proteins mCh-SH3b2, mCh-SH3b6, mCh-E472A, mCh-E472V, mCh-G501A, mCh-G501D, mCh-D503A, mCh-W508A, as well as mCh-SH3b2ΔC2, mCh-SH3b6::C2. Next, cells were washed twice with warm media to remove extracellular protein and fixed with 4% paraformaldehyde for 20 min with mild agitation. Cells were washed with 1% PBS and quenched with 1% ammonium chloride (NH_4_Cl) solution prepared in PBS for 10 min with agitation. After washing with PBS, cells were treated with 1 μg/mL wheat germ agglutinin (WGA) Alexa Fluor 488 (Invitrogen™, Thermo Scientific, USA) for 20 min with agitation, followed by washing with PBS. Next, cells were treated with 1 μg/mL of DapI (Thermo Scientific™, USA) prepared in PBS for 20 min with mild agitation. Cells were washed with PBS before mounting on glass slides using ProLong™ Gold Antifade Mountant (Invitrogen™, Thermo Scientific, USA). Slides were kept in the dark for 24 h before imaging using Leica Stellaris DMI8 confocal microscope at 63x magnification with numerical aperture 1.40 oil. The confocal pinhole was maintained at 1.0 Airy unit. Fluorophores were excited sequentially to minimize crosstalk using 405nm, 488nm, and 561 nm laser lines for DAPI, WGA and mCherry, respectively. Laser intensity was kept constant at 2%, and images were captured at 8-bit depth for all imaging sessions. Images were deconvolved, and Z-stacks were made by Leica LAS X (version 4x) using Lightning mode. Z-step size was kept between 0.2-0.3 with 25–35 steps. Cross-sections of the Z-stacks were generated using the Sections option; the region of the stack to be displayed was defined manually in Slice view using the Select Range option, and cross-sectional images were then exported with the Extract option. Stitched images were created by navigating the large area of at least three slides per experiment using the LAS X navigator. Images were captured from at least three independent experiments per condition tested. For quantification, 15 images from the stitch were analyzed using FIJI/Image J software. First, the image was split into separate channels, and the background was removed using the Subtract Background command from the red channel. Integrated density of the mCherry signal was measured inside the cells using the Measure command in the Analyze tab. Cell nuclei were counted manually from each image. Mean fluorescence intensity per cell (AU) was calculated by dividing the integrated density by the number of cells for each image. One-way analysis of variance (ANOVA) and *post-hoc* Tukey test were performed using all values obtained for Mean fluorescence intensity (AU). Different letters on top of the bars represent statistical significance at <0.05.

### 2.11 Resazurin reduction assay

Cell viability of Mp in response to the protein treatment was determined using resazurin reduction assay. Mp were seeded in a 96 well plate at 6.00E + 3 cells/well and incubated at 37 ˚C and 5% CO_2_ to reach confluence. Cells were treated with either 1.5 µg/mL or 5 µg/mL proteins for 18 hours in triplicates. After the incubation, cells were washed with fresh media and incubated for 4 h with Resazurin sodium salt (Acros Organics, Thermo Fisher, MA, USA) at a final concentration of 15 µg/mL prepared in media with no phenol red. After incubation, raw fluorescence units (RFU) were measured using Agilent BioTek Synergy H1 plate reader with default resazurin fluorophore settings (Agilent, CA, USA). Media alone was used as blank and untreated cells were used as a positive control. Cell viability was expressed as percentage relative to untreated cells.

### 2.12 Statistical analyses

One-way analysis of variance (ANOVA) and *post hoc* Tukey test were performed on three biological replicates. Different letters on top of the bars denote statistical significance <0.05.

## 3 Results

### 3.1 SH3b domains of Sdp differentially stimulate IRF3/IRF9, NF-ĸB/AP-1 pathways in macrophages.

In a previous study, we reported that the SH3b2 domain of Sdp was the only EVs enriched protein domain, of the tested proteins, that stimulated cellular innate immune signaling both *in vitro* and *in vivo* (8, 21). SH3b2 and EVs equally induced the promoters of NF-ĸB and IRF pathways in RAW DUAL™ KO-TLR4 Mp, while other proteins purified from EVs, as well as the SH3b6 domain of Sdp did not induce the promoters of both pathways (21). Sdp consists of six SH3b domains, in which five domains have highly conserved sequences that differ in a small number of residues, while SH3b6 showed more sequence divergence (**Supplementary Figure S2C**) (8). First, we sought to investigate the innate immune response stimulation properties of the four remaining domains (SH3b1, SH3b3, SH3b4 and SH3b5) compared with SH3b2 and SH3b6. We utilized RAW-Lucia™ ISG Cells, RAW-Blue™ Cells and RAW DUAL™ KO-TLR4 Mp reporter cell lines.

The purified SH3b domains (**Supplementary Figure S1**) were evaluated at 1.5 μg/mL. LPS (1 µg/mL) served as a negative control for RAW DUAL™ KO-TLR4 Mp and as a positive control for WT Mp cell lines as it is a TLR4 agonist. 2’3’ cGAMP (5 µg/mL) was used as a positive control for ISRE promoter induction. Media alone was used as a negative control for all assays. It was found that the SH3b2 domain significantly induced the promoters of NF-ĸB/AP-1 as well as IRF3/IRF9 pathways in all Mp cell lines tested (**Figures 1A–D**), which is consistent with our previous findings (21). A similarly significant level of induction of both promoters was observed for the SH3b1 domain. However, the ability of SH3b3, SH3b4, and SH3b5 to induce the expression of the MIP-2 and ISRE promoters was significantly lower, and their stimulation was dependent on the cell line and the promoter tested. The addition of SH3b6 only showed activation of both promoters in RAW Blue cell line, albeit at very low levels, in agreement with our previous report (21). We hypothesize that the difference in activity of the promoters tested is related to the few variable residues found between the SH3b domains in Sdp. These residues may selectively be involved in interactions with host proteins involved in uptake or trafficking of the SH3b domains into the host cell.

**Figure 1:**
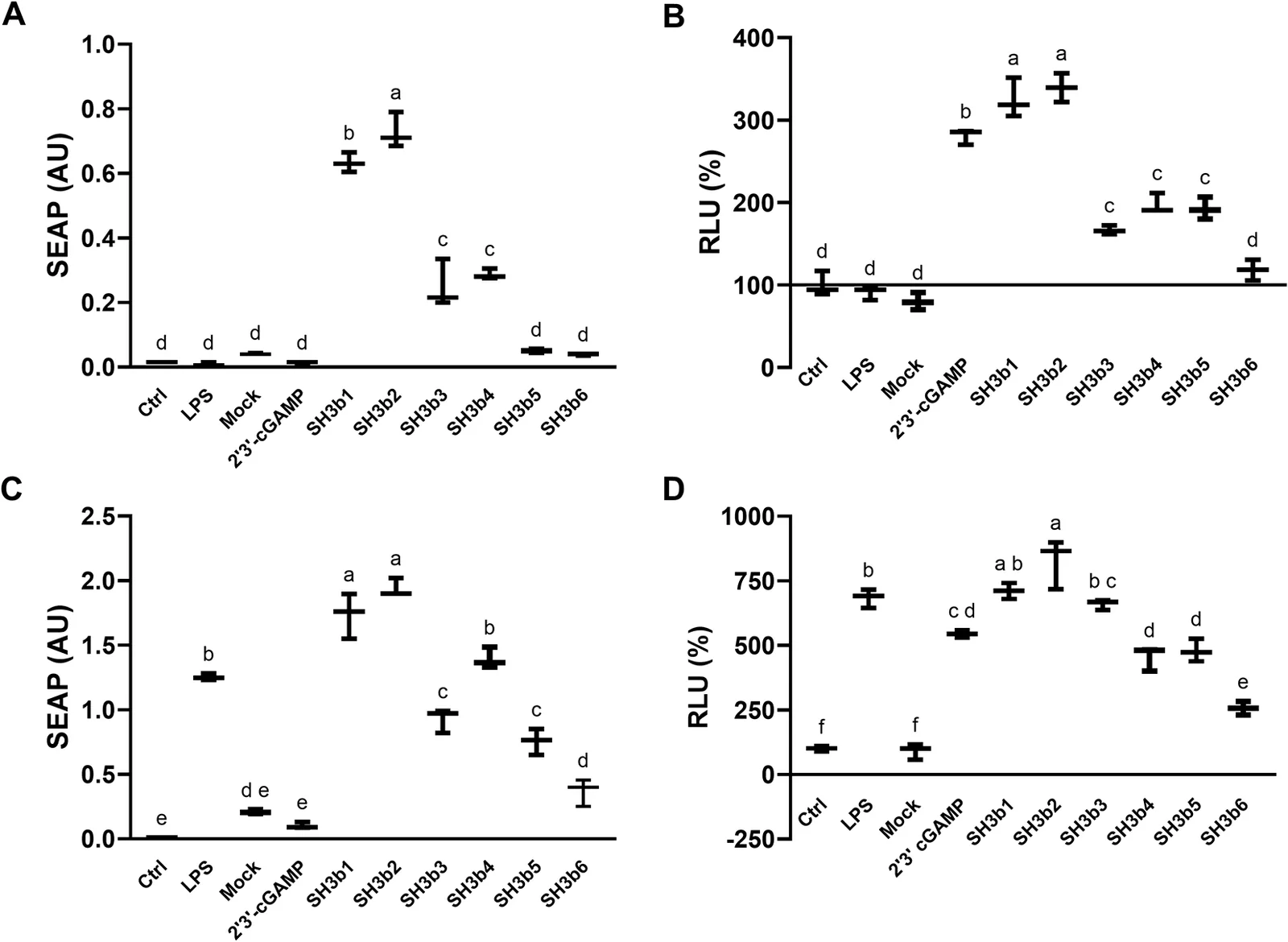
SH3b domains of Sdp induce NF-ĸB/AP-1 and IRF pathways in RAW DUAL™ KO-TLR4 **(A, B)**, RAW-Blue™ **(C)** and RAW-Lucia™ ISG **(D)** Mp reporter cell lines. **(A, C)** MIP-2 and **(B, D)** ISRE promoter activity was quantified by measuring SEAP and Luc activity, respectively. Mp cell lines were treated with 1.5 μg/mL purified Sh3b domains (SH3b1, SH3b2, SH3b3, SH3b4, SH3b5 & SH3b6) for 18 h, followed by SEAP **(A)** and Luc assays **(B)**. Cell supernatant without any treatment (Ctrl) was used as a blank control; LPS was used as a negative control for RAW DUAL™ KO-TLR4 Mp cells and as a positive control for WT Mp cells; 2’3’ cGAMP was utilized as a negative control for SEAP activity induction and a positive control for ISRE activity. Different letters above bars represent statistically significant differences at p ≤ 0.05 based on one-way ANOVA followed by Tukey’s *post-hoc* test.

### 3.2 Structural modeling revealed residues in SH3b2 potentially involved in host signal transduction

SH3b domains are homologs of eukaryotic SH3 domains, which trigger diverse cell signaling pathways. Initiation of signaling pathways is mostly driven by interaction of specific residues in SH3 domains with the proline-rich motif or PxxP (where x is any amino acid) motif of other cellular proteins (28, 35, 48). To evaluate whether SH3b2 immune stimulation is mediated through interactions with proline-rich regions of host proteins, first we performed structural modeling of SH3b2. Using Phyre 2.2 in standard settings, identified a hydrolase from *Staphylococcus* phage G15 domain (PDB: 2MK5, (49)) that was used as a template. SH3b domains largely exhibit a β-barrel fold with multiple β strands, approximately eight in groups forming two β sheets (33, 34, 50). The modeled SH3b2 predicted a slightly different structure comprised of nine β strands grouped in three β sheets, where β2, β5, and β9 form sheet A; β3 and β4 form sheet B; and β1, β6, β7, and β8 form sheet C (**Supplementary Figure S2A**). Next, we superimposed SH3b2 predicted model with eukaryotic SH3 domains whose molecular structure was obtained in the presence of proline-rich sequences and have been characterized to interact with proline-rich motifs.

Structural alignment of the SH3b2 model was performed with chain A of cortactin (PDB: 2D1X, (42)) and the N-terminal SH3 (N-SH3) domain of growth factor receptor-bound protein 2 (Grb2) (PDB: 1AZE, (43)) (42, 43). Cortactin interacts with the proline-rich peptide (KRPPPPPPG) of AMAP-1, while Grb2 binds to the proline-rich peptides of dynamin (GPPPQVPSRPNR) and SOS (VPPPVPPRRR) proteins (42, 43). Structural alignment of the SH3b2 model and selected eukaryotic SH3 domains was performed using the UCSF ChimeraX 1.9 structural analysis matchmaker tool. The use of Smith-Waterman Sequence alignment algorithm with Blossom 35 matrix settings for the structural alignment of SH3b2 with Grb2 N-SH3, retrieved a sequence alignment score of 48.8. For the structural alignment of SH3b2 and Cortactin, the Needleman-Wunsch alignment algorithm with the BLOSUM50 matrix was utilized, with an alignment score of 30.1.

Next, residues in SH3b2 that align with Grb2 N-SH3 or Cortactin and lie within 4Å radius of the proline-rich peptides of AMAP-1 or SOS, respectively, were identified. These residues in SH3b2 were predicted to be important for SH3b2 immunostimulatory properties through protein/protein interactions in macrophages. The residues N470, E472, G501, D503, Q506, W508 and T510 in the SH3b2 domain (amino acid numbers are relative to the full-length Sdp protein) were selected for site-directed mutagenesis. All the residues were initially mutated to alanine (**Figures 2A, B**).

**Figure 2:**
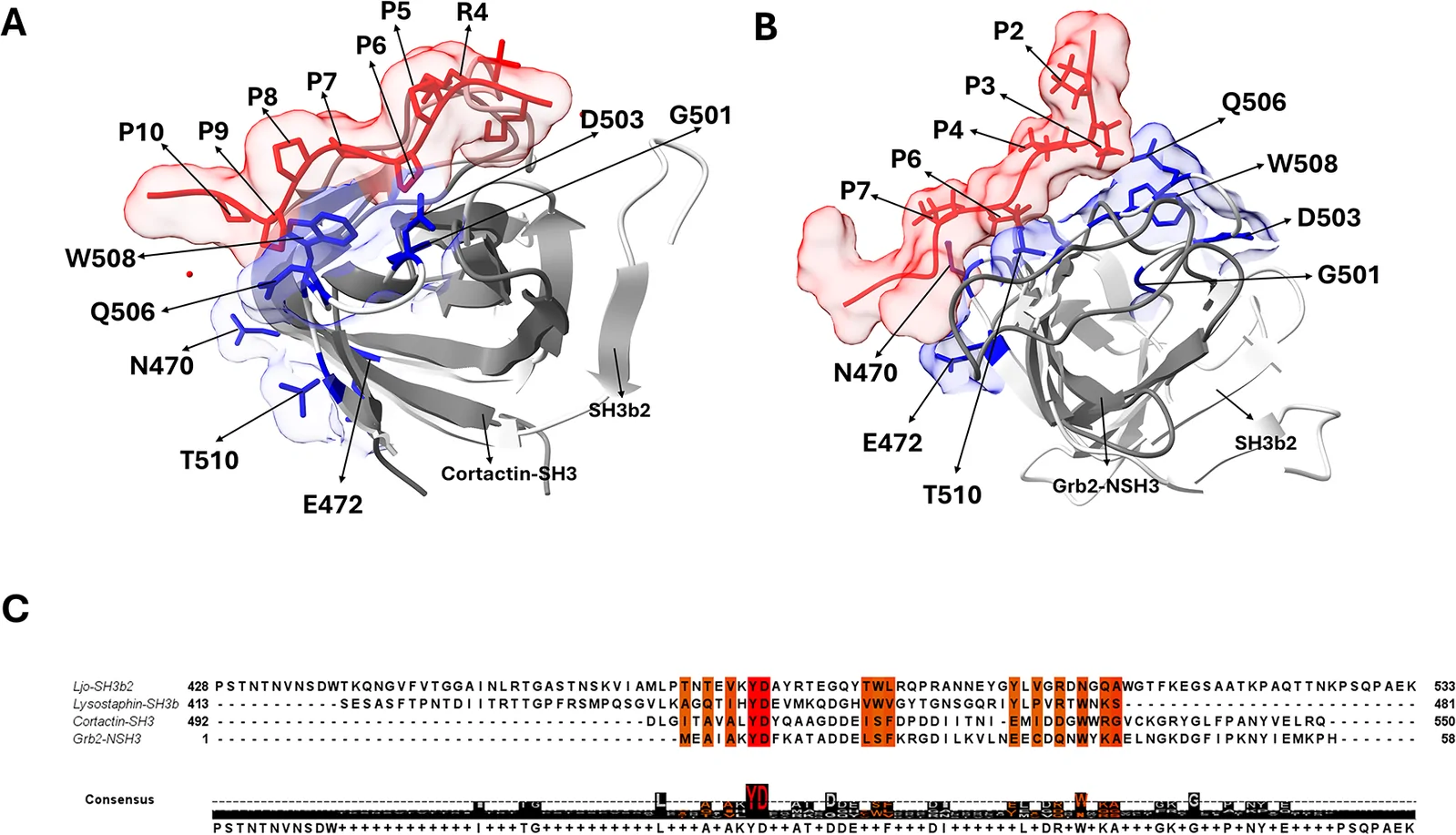
Structural alignment identifies putative residues in SH3b2 involved in interactions with proline-rich motifs. **(A)** Structural alignment of Cortactin SH3 domain (PDB: 2D1X), dark gray, or **(B)** Grb2 NSH3 domain (PDB: 1AZE), dark gray, with the model of SH3b2, light gray. **(A, B)** The proline-rich motif is shown in Red. Residues in SH3b2 within 4Å radius of the proline-rich sequence are shown in blue. Structural alignment was performed using the ChimeraX pairwise alignment tool. Images were adjusted and edited using ChimeraX. **(C)** Multisequence alignment of SH3 and Sh3b domains performed with MultAlin and Jalview tools (39, 40).

We also set out to identify the key structural differences between SH3b2 and SH3b6, as SH3b6 consistently did not induce the MIP-2 and ISRE promoters in all cell lines tested. Multisequence alignments of SH3b domains showed that SH3b6 has a shorter sequence compared to all SH3b domains of Sdp. It shares a 54.2% identity to Sh3b2, with differing residues at various locations (**Supplementary Figure 2C**). We performed structural modeling of SH3b6 using Phyre2.2 using hydrolase from *Staphylococcus simulans* (PDB: 4LXC (51),) found as the best template. The predicted model of SH3b6 consists of eight β strands where β1, β4, and β8 are grouped together forming sheet A, while β2 and β3 form sheet B, and β5, β6, and β7 make sheet C (**Supplementary Figure S2B**). The predicted model of SH3b6 has distinct features compared to SH3b2, indicating that further analysis is required to determine whether these differences prevent SH3b6 internalization into cells or its interaction with other proteins within Mp cells.

### 3.3 Single amino acid replacement altered the immunostimulatory effect of SH3b2

To identify the specific amino acids in SH3b2 involved in SH3b2-mediated signal transduction, the purified SH3b2 mutant proteins (**Supplementary Figure S3**) were evaluated using RAW DUAL™ KO-TLR4 Mp. It was found that SH3b2 mutants D503A, Q506A, and G501A had a comparable MIP-2 induction activity to the WT SH3b2 (**Figure 3A**). The activity of the MIP-2 promoter was significantly reduced when N470A and T510A were tested, while the mutation W508A, resulted in an inactive SH3b2 protein, and no stimulation of the MIP-2 promoter was observed. In contrast, a significant induction of MIP-2 was observed with E472A compared to WT SH3b2 (**Figure 3A**).

**Figure 3:**
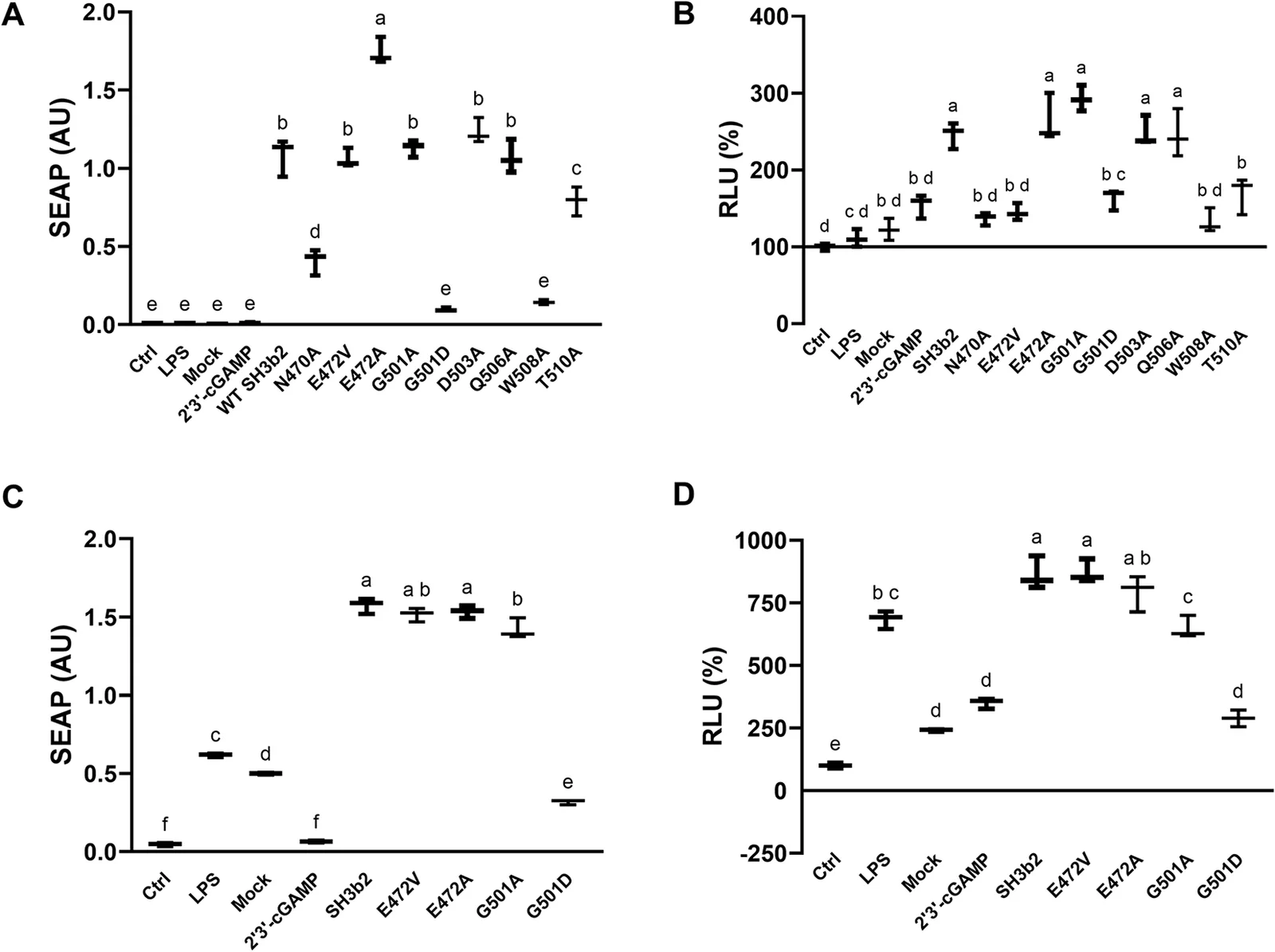
Mutations in SH3b2 differentially impact the induction of the MIP-2 and ISRE promoter activity in reporter Mp cells to activate NF-ĸB/AP-1 and IRF pathways. **(A, B)** Purified SH3b2 WT or mutant versions N470A, E472V, E472A, G501A, G501D, D503A, Q506A, W508A, T510A were tested in RAW DUAL™ KO-TLR4. WT SH3b2, E472A, E472V, G501A and G501D were tested on RAW-Blue™ **(C)** and RAW-Lucia™ ISG **(D)** Mp reporter cell lines. Mp cell lines were treated with 1.5 μg/mL of purified proteins for 18 h followed by SEAP **(A, C)** and Luc assays **(B, D)**. Cell supernatant without any treatment (Ctrl) was used as a blank control; LPS was used as a negative control for RAW DUAL™ KO-TLR4 Mp cells and as a positive control for WT Mp cells; 2’3’ cGAMP was utilized as a negative control for SEAP activity induction and a positive control for ISRE activity. Different letters above bars represent statistically significant differences at p ≤ 0.05 based on one-way ANOVA followed by Tukey’s *post-hoc* test.

To determine whether replacing some residues originally mutated to alanine with a charged amino acid, aspartic acid (D), affects protein activity, G501 was replaced with D (G501D). In addition, we replaced E472 with valine (V), as this residue is found in the inactive SH3b6. It was found that E472V had no effect on the stimulatory activity of SH3b2 on the MIP-2 promoter, while G501D resulted in an inactive SH3b2 protein. Analyses of the ISRE promoter showed a similar trend to MIP-2, with a significant reduction in promoter activity by W508A and G501D. However, N470A and E472V treatment of Mp also significantly reduced the ISRE promoter activity. ISRE promoter was significantly induced by E472A and G501A (**Figure 3B**), when compared to WT SH3b2. To verify whether site directed mutagenesis resulted in misfolded proteins, differential scanning fluorimetry (DSF) was performed. It was found that WT SH3b2 and the mutants, G501D and W508A, have similar melting temperatures (Tm) (**Supplementary Figure S4**). These results suggest that mutations in SH3b2 did not cause major folding disruptions.

The results obtained with SH3b2 mutants G501A, G501D, E472A, and E472V in RAW DUAL™ KO-TLR4 Mp were validated in wild-type RAW-Lucia™ ISG and RAW-Blue™ cell lines. We observed that G501D significantly reduced MIP-2 and ISRE promoter stimulation in both cell lines, while other mutants tested had a similar level of promoter induction as WT SH3b2 (**Figures 3C, D**).

To evaluate whether the addition of SH3b2 or mutant proteins had a cytotoxic effect on Mp, the resazurin reduction assay (RDA) was performed. Cell viability was determined after 18 h of treatment with 1.5 and 5 µg/mL of WT SH3b2 or the mutant versions. No significant effects of the treatments on Mp viability were found when compared to untreated Mp (**Supplementary Figures S5A, B**).

### 3.4 Specific mutations altered the SH3b2-mediated MNV-1 antiviral response

We previously reported that EVs and SH3b2 reduced the replication of murine norovirus (MNV-1) both *in vitro* and *in vivo* (21). The treatment with EVs and SH3b2 in RAW 264.7 Mp resulted in a significant induction of *Oas1*b antiviral genes as well as the tolerogenic *IL10* gene expression (21). Next, it was evaluated whether specific mutations in SH3b2 altered its ability to induce antiviral response and reduced MNV-1 RNA levels. For this experiment, mutant SH3b2 proteins were selected based on their ability to induce MIP-2 and ISRE promoter activity. RAW 264.7 Mp cells were treated with either a media-only control, WT SH3b2, or the selected mutants, including G501A, G501D, D503A, and W508A, for 6 h. After incubation, one set of cells was challenged with MNV-1 (MOI = 5) for 1 h, while the other set of cells remained uninfected. The expression of *Oas1*b and *IL10* was measured 6 h post-infection (hpi), while the MNV-1 genome was quantified by qPCR at 18 hpi as previously reported (21).

It was observed that WT SH3b2, G501A, and D503A significantly induced *Oas1*b and *IL10* gene expression in uninfected RAW 264.7 Mp. Whereas a significant reduction in gene expression was observed in the Mp treated with G501D and W508A (**Figures 4A, B**). In MNV-1 infected cells, WT SH3b2 and G501A treatment significantly induced the expression of *Oas1*b and *IL10* genes which was correlated to a significant reduction in MNV-1 RNA levels. In contrast, the mutation in G501D abolished the induction of the expression of *Oas1*b and *IL10* genes resulting in a high RNA abundance of MNV-1, similar to untreated cells (**Figures 4C–E**). These results indicate that G501 plays an important role in interactions of SH3b2 with host proteins contributing to SH3b2 domain immunostimulatory response.

**Figure 4:**
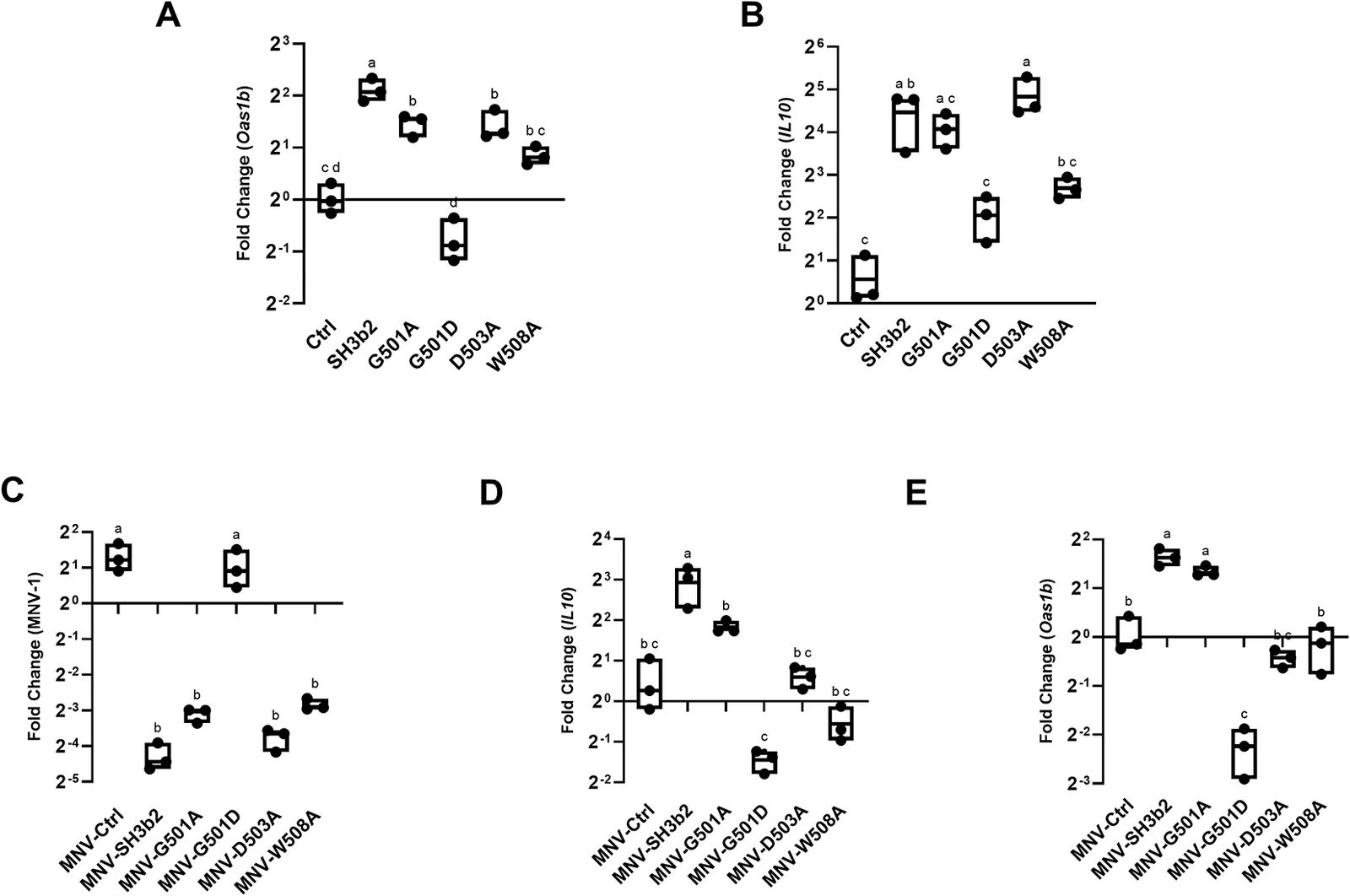
Specific residues in SH3b2 reduce MNV-1 infection in RAW 264.7 Mp cells and induce *Oas1b* and *IL10* mRNA expression. RAW 264.7 cells were treated with 1.5 µg/mL of SH3b2, G501A, G501D, D503A, and W508A for 6 h prior to MNV-1 infection. Uninfected Mp were analyzed for *Oas1b* and *IL10* expressions **(A, B)**, while infected cells were assessed for *Oas1b*, *IL10*, and *MNV-1* expression **(C–E)** by qRT-PCR. Different letters above bars represent statistically significant differences at p ≤ 0.05 based on one-way ANOVA followed by Tukey’s *post hoc* test. Three biological replicates and two technical replicates per qRT-PCR reaction were included in the data analysis.

### 3.5 WT SH3b2 and mutants show comparable internalization by THP-1 macrophages

To determine whether mutations in SH3b2 may affect SH3b2 internalization within the THP-1 cells, SH3b2 was fused to the C-terminus of the mCherry fluorescent protein. Cellular uptake was visualized and quantified by confocal microscopy. PMA activated THP-1 Mp were treated with 5 μg/mL of the mCherry fusion proteins for 1 h. Untreated cells and cells treated with purified mCherry were used as a negative or specificity control, respectively. Confocal microscopy indicated that mCh-SH3b2 and mCh-SH3b2 mutants can be taken up by THP-1 Mp regardless of the mutation present. Quantitative analysis revealed significantly higher mCherry signal with the mCh-G501A, mCh-E472A, and mCh-E472V mutants compared to mCh-SH3b2. In contrast, lower mCherry signal was observed with the mCh-G501D, mCh-D503A, and mCh-W508A treatments compared to mCh-SH3b2. No significant uptake into THP-1 Mp was detected using mCherry control (mCh) (**Figures 5A–C**). In summary, despite different levels of mCherry signal, all the SH3b2 mutants were internalized by THP-1 macrophages. Representative images via orthogonal sectioning of Z-stacks depict the internal localization of SH3b2 as well as the G501D and W508A mutants (**Figure 6**). These results suggest that interactions of SH3b2 with specific intracellular proteins are likely responsible for activation of the sensory pathway while the mutations introduced do not largely impact the uptake of the protein into the host cell.

**Figure 5:**
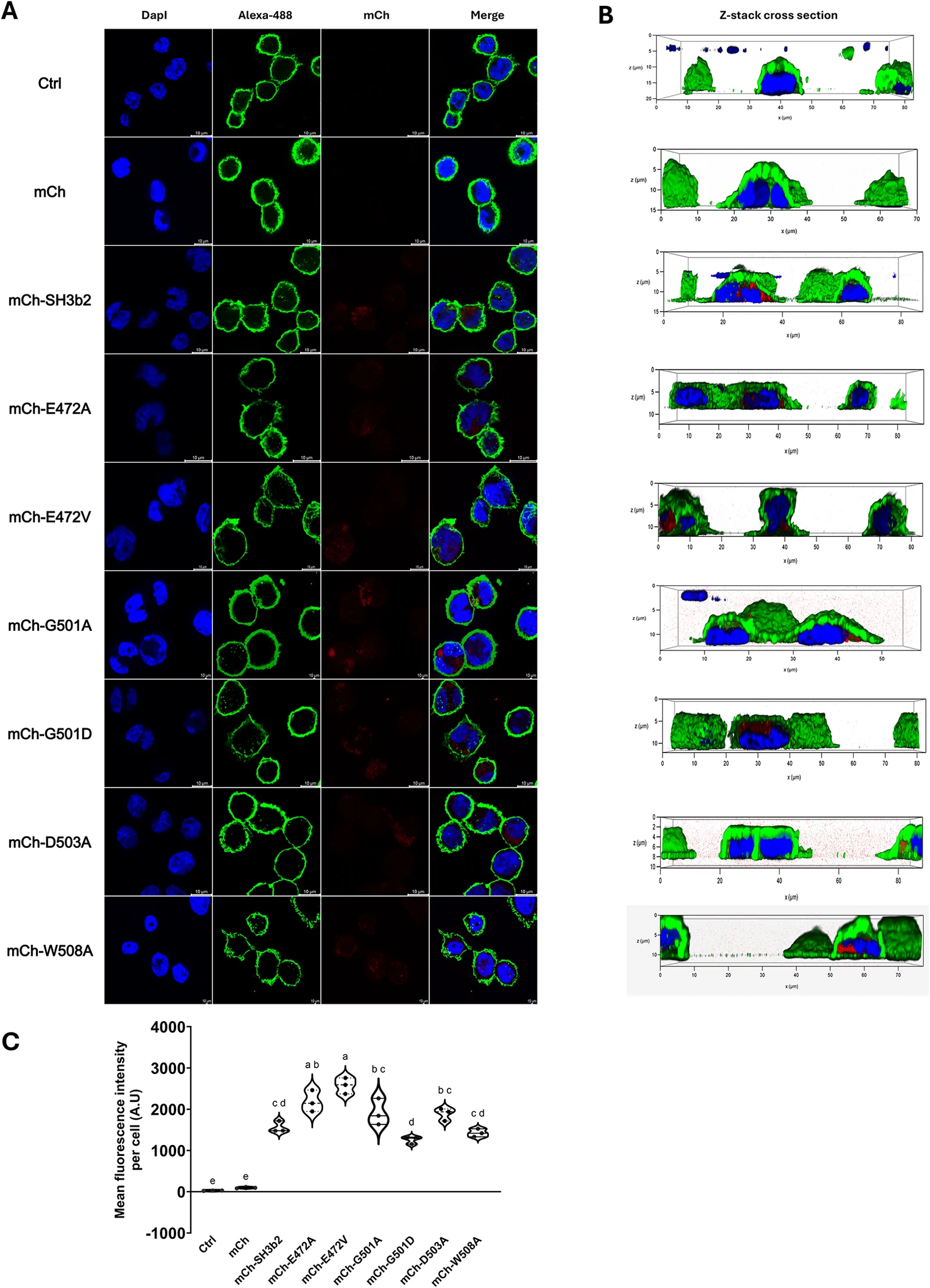
Mutations on SH3b2 do not significantly impact uptake by THP-1 Mp. Representative confocal microscopy images were obtained using SH3b2 fusions to the C-terminus of mCherry (mCh). Mp cells were treated with 5 µg/mL of either purified mCh or mCh-SH3b2, mCh-E472A, mCh-E472V, mCh-G501A, mCh-G501D, mCh-D503A, mCh-W508A for 1 h followed by slide preparation as discussed in the methodology section. **(A)** Staining was performed for THP-1 nuclei (DAPI, Blue), THP-1 cell membrane (Alexa488, Green), and mCherry fusion proteins (Red), as indicated at the top of the panels. The protein used in each set is indicated on the left of the images. **(B)** Representative z-stacks obtained for the mCherry fusion protein. **(C)** Quantitative assessment of mean fluorescent intensity (AU) per cell was calculated by dividing the integrated density of the red channel by the number of cells in each image (stitched images are available as **Supplementary Figure S4**). Different letters above bars represent statistically significant differences at p ≤ 0.05 based on one-way ANOVA followed by Tukey’s *post-hoc* test.

**Figure 6:**
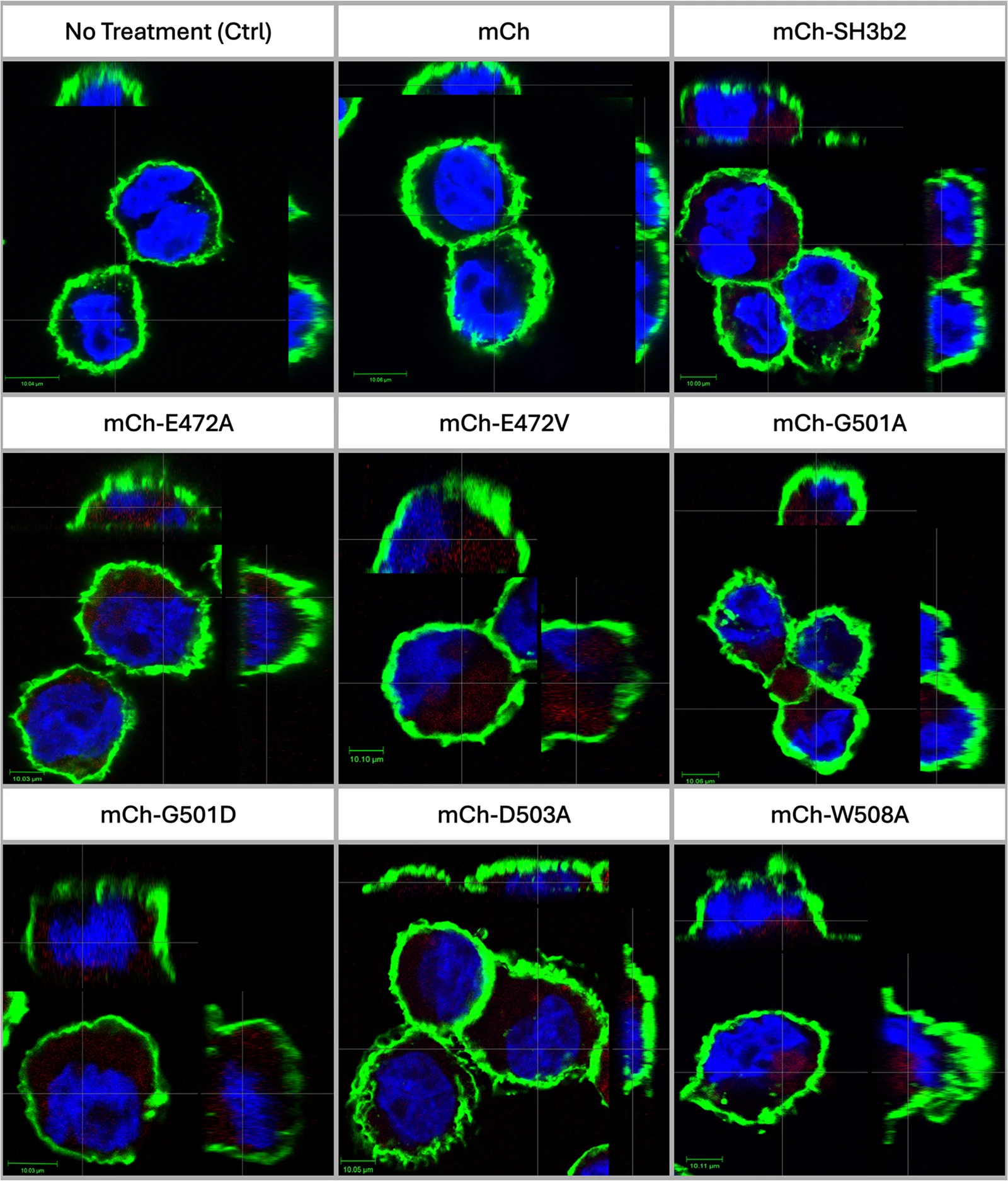
Representative images obtained via orthogonal sectioning of Z-stacks depicts the internal localization of SH3b2 proteins. Z-stack orthogonal sections were obtained using SH3b2 fusions to the C-terminal of mCherry (mCh). Mp cells were treated with 5 µg/mL of either purified mCh or mCh-SH3b2, mCh-E472A, mCh-E472V, mCh-G501A, mCh-G501D, mCh-D503A, mCh-W508A for 1 h, followed by slide preparation as discussed in the methodology section. Staining was performed for THP-1 nuclei (DAPI, Blue), THP-1 cell membrane (Alexa488, Green, WGA), mCherry fusion proteins (Red). Z-step size was set between 0.2-0.3 with 25–35 steps for all images. orthogonal sectioning was captured using Leica LAS X (version 4x) Orthogonal Sections tool for Z-stacks.

### 3.6 The C-terminal linker sequence has a significant contribution to the role of SH3b2 in host cell signaling

To identify whether the C-terminal linker sequence between SH3b domains plays a role in protein uptake by Mp cells, we first created recombinant proteins SH3b2ΔC2 and SH3b6::C2. It was found that the addition of the C-terminal linker sequence of SH3b2 to SH3b6 (SH3b6::C2) was able to partially induce the stimulation of the MIP-2 and ISRE promoters when compared to the WT SH3b2. In contrast, the deletion of the C-terminal linker sequence in SH3b2 (SH3b2ΔC2) resulted in significantly decreased induction of the reporter promoter (**Figures 7A–D**). Based on these findings, we hypothesized that the C-terminal linker sequence is partially involved in the internalization of SH3b domains by Mp cells.

**Figure 7:**
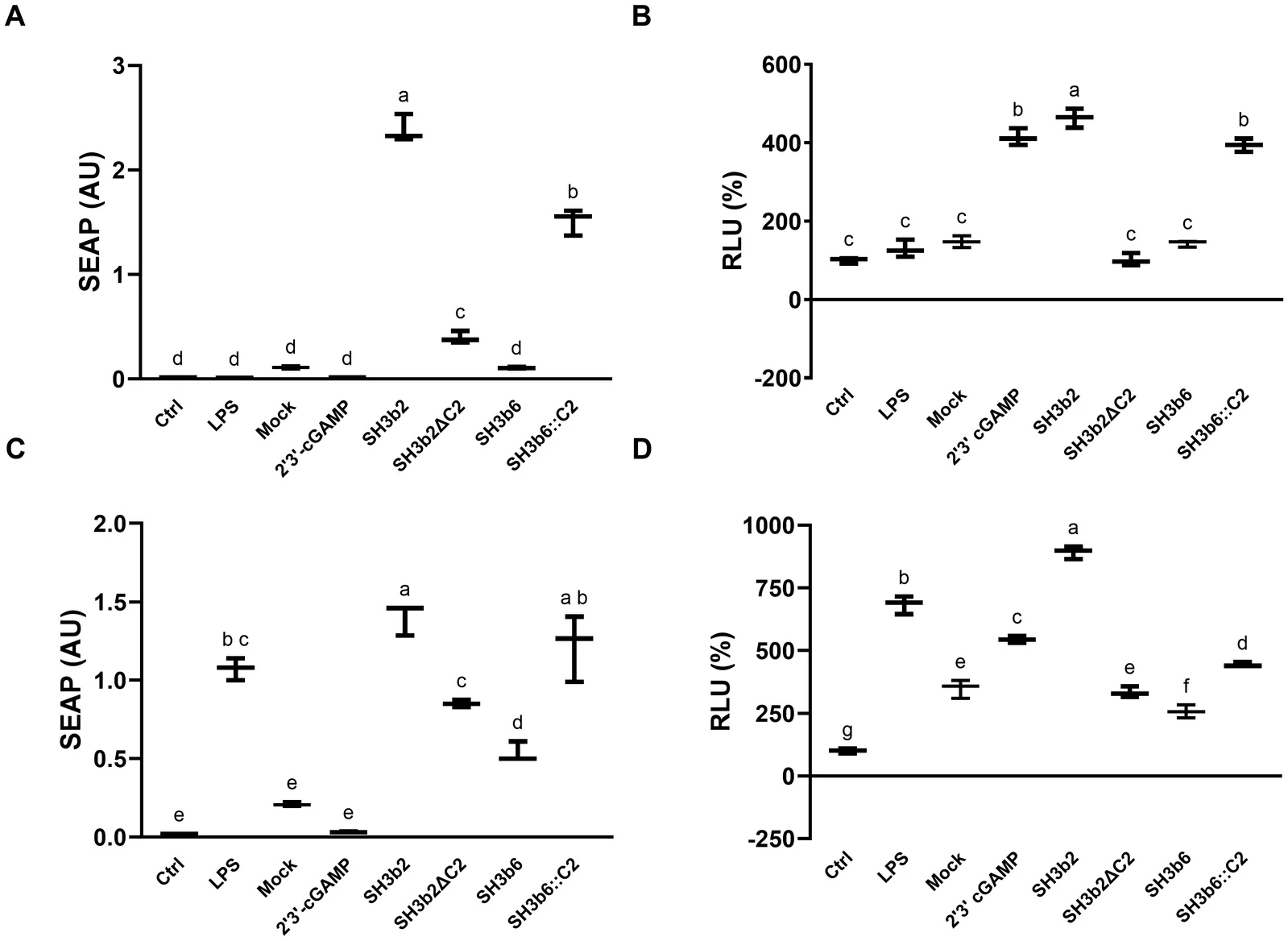
The C-terminal linker sequence has a partial role in SH3b2-derived cell signaling. Mp cell lines RAW DUAL™ KO-TLR4 **(A, B)**, RAW-Blue™ **(C)** and RAW-Lucia™ ISG **(D)** Mp reporter cell lines were treated with 1.5 μg/mL of SH3b2, SH3b6, SH3b2ΔC2, and SH3b6::C2 proteins for 18 h followed by SEAP **(A, C)** and Luc assays **(B, D)**. Cell supernatant without any treatment (Ctrl) was used as a blank control; LPS was used as a negative control for RAW DUAL™ KO-TLR4 Mp cells and as a positive control for WT Mp cells; 2’3’ cGAMP was utilized as a negative control for SEAP activity induction and a positive control for ISRE activity. Different letters above bars represent statistically significant differences at p ≤ 0.05 based on one-way ANOVA followed by Tukey’s *post-hoc* test.

To test this hypothesis, we fused mCherry fluorescent proteins with the recombinant SH3b2ΔC2 and SH3b6::C2 proteins as well as with the SH3b6 domain. Visualization via confocal microscopy revealed significant differences in uptake of mCh-fusion proteins depending on the presence of the C-terminal linker sequence (**Figures 8A–C**). While the treatment of Mp cells with mCh-SH3b6 resulted in very low uptake, the addition of the C-terminal of SH3b2 to SH3b6 (SH3b6::C2) increased the level of uptake, albeit it was significantly lower than SH3b2. The deletion of the C-terminus of SH3b2 (SH3b2ΔC2) resulted in significantly reduced levels of uptake (**Figures 8A–C**; **Supplementary Figure S7****).** These results indicate that the C-terminal linker plays a partial role in the SH3b domain’s cell signaling activity, and it is not the only factor driving the internalization of the SH3b domain within the macrophages.

**Figure 8:**
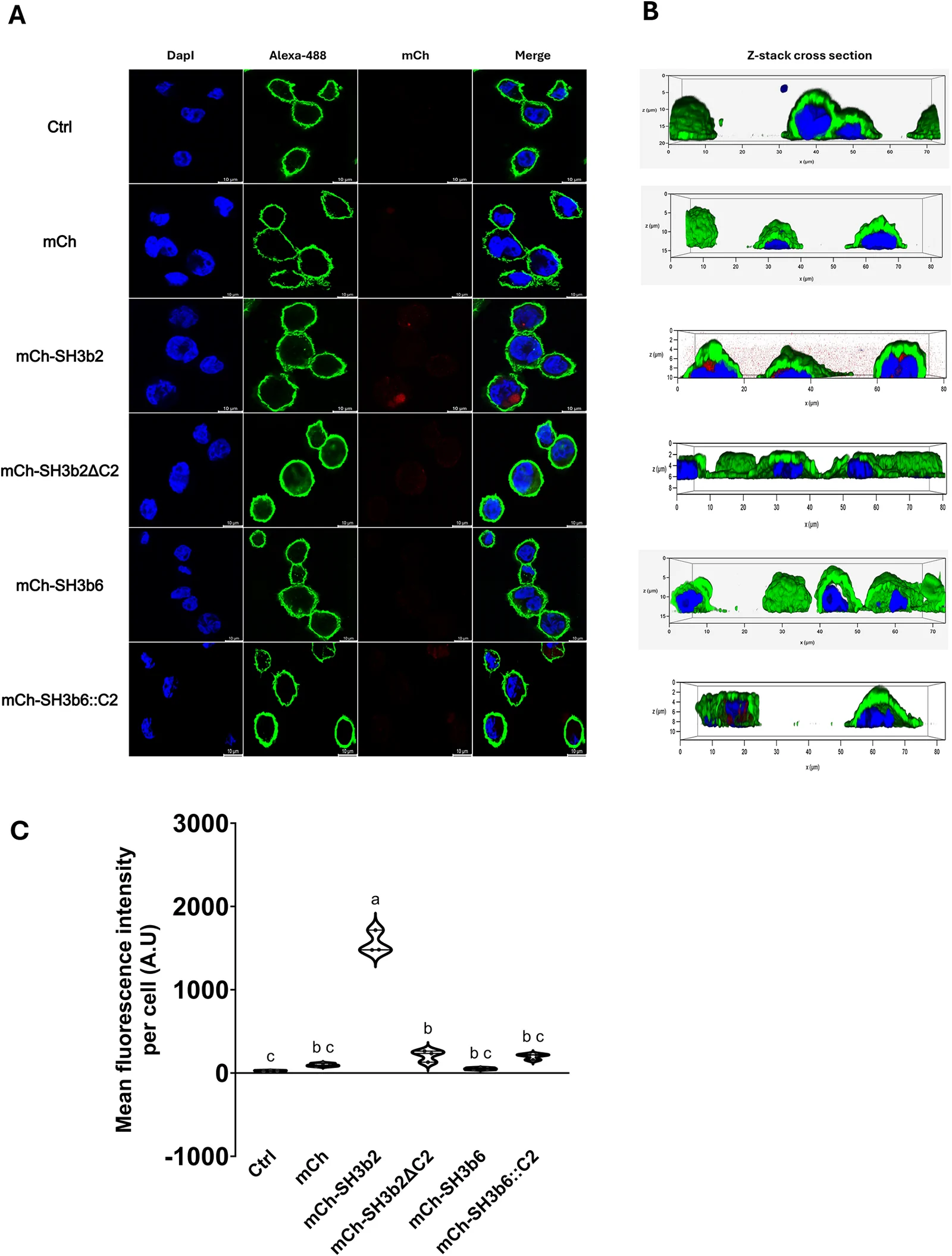
The C-terminal linker sequence is required for uptake of SH3b2 by Mp. Representative confocal microscopy images were obtained using SH3b2 fusions to the C-terminal of mCherry (mCh). Mp cells were treated with 5 μg/mL of either purified mCh or mCh-SH3b2, mCh-SH3b2ΔC2, mCh-SH3b6::C2, and mCh-SH3b6 for 1 h, followed by slide preparation as discussed in the methodology section. **(A)** Staining was performed for THP-1 nuclei (DAPI, Blue), THP-1 cell membrane (Alexa488, Green), mCherry fusion proteins (Red), as indicated at the top of the panels. The protein used in each set is indicated on the left of the images. **(B)** Representative Z-stacks obtained for the mCherry fusion protein. **(C)** Quantitative assessment of mean fluorescent intensity (AU) per cell was calculated by dividing the integrated density of red channel with the number of cells in each image (stitched images are available as **Supplementary Figure S4**). Different letters above bars represent statistically significant differences at p ≤ 0.05 based on one-way ANOVA followed by Tukey’s *post-hoc* test.

## 4 Discussion

The discovery of defined bacterial effector molecules that govern host-microbe signaling represents a critical advance, moving the field beyond generic probiotic supplementation toward the rational design of precision, biomimetic therapeutics. In this study, we advance our understanding of the mechanisms used by SH3b domains contained in extracellular vesicles from *L. johnsonii* N6.2. We showed that despite high sequence conservation across five of the six internal Src homology 3 (SH3b1-5) domains of the Sdp protein (8), these domains exhibit markedly unequal immunostimulatory capacities. While SH3b2 and SH3b1 robustly stimulated both the NF-κB/AP-1 and IRF pathways in macrophages, domains SH3b3–5 exhibited markedly lower, cell-line-dependent activation. The divergent SH3b6 domain remained largely inert. The phenomenon where highly conserved, tandemly repeated bacterial domain architectures exhibit vastly different signaling outcomes has been documented across multiple surface-layer and cell-wall-anchored proteins. For example, similar functional compartmentalization is observed in the multi-domain internalin proteins of *Listeria monocytogenes* (52) and the repeat-domain structures of *Staphylococcus aureus* fibronectin-binding proteins, FnBPA and FnBPB (53, 54), where minor sequence alterations alter host receptor affinity.

The structural compartmentalization observed in SH3b suggests that *L. johnsonii* has evolved highly specialized functional regions within a single surface protein to interact with the host. Our structural modeling suggests that this functional dichotomy is driven by a sophisticated strategy of cross-kingdom molecular mimicry. While bacterial SH3b domains typically feature an eight-stranded β-barrel fold designed to recognize bacterial cell-wall peptidoglycans, our structural alignment revealed that SH3b2 potentially adopts a unique, modified nine-stranded orientation. This structural topology closely mimics eukaryotic adapter architectures, such as Cortactin and the N-terminal SH3 domain of growth factor receptor-bound protein 2 (Grb2). Eukaryotic SH3 domains are classical mediators of signal transduction, operating by anchoring onto proline-rich (PxxP) motifs of downstream host intracellular kinases or regulatory proteins (27, 43, 55). Superimposing the SH3b2 model with the crystal structures of Cortactin and Grb2 complexed with their respective proline-rich target peptides (AMAP-1 and SOS/dynamin), we predicted the spatial orientation of the *L. johnsonii* effector. Using this structure-guided site-directed mutagenesis, we identified key residues that can either enhance the cellular signaling or halt it, either partially or completely. These results indicate that *L. johnsonii* has evolved a structural analog capable of interacting with conserved host eukaryotic signaling cascades, a strategy described for a variety of pathogenic microorganisms (56).

Molecular mimicry of host SH3/PxxP- interaction networks has been proposed as a weapon utilized by pathogens such as *E. coli and Mycobacterium tuberculosis* to disrupt, hijack, or paralyze host cells. EspF from enteropathogenic *E. coli* has been extensively documented to bind SH3 domains through the C-terminal eukaryotic-like proline-rich repeats (PRR) (57–59). On the other side of the interaction dyad, *Mycobacterium* PPE15 contains an SH3e domain that structurally resembles eukaryotic adapters that interact with N-WASP and the NADPH oxidase (NOX) complex, thereby inhibiting NOX complex assembly and the generation of reactive oxygen species (59). In contrast, our work highlights a counterexample. We propose that by being actively taken up by macrophages and utilizing a eukaryotic-like protein interface, the SH3b2 domain acts as a multivalent regulatory hub. It stimulates innate immune cascades by activating IRF-dependent antiviral pathways (such as *Oas1*), while simultaneously co-activating homeostatic, anti-inflammatory responses (such as IL-10). This dual-pathway engagement provides a balanced, highly protective immune profile that avoids the tissue-damaging over-activation often associated with viral sensing networks.

The best-characterized role of SH3b domains is their role in peptidoglycan binding, as reported for the C-terminal SH3b domain of lysostaphin from *Staphylococcus* (34, 35, 51, 60–62). Lysostaphin is a potent bacteriolytic endopeptidase that relies strictly on its SH3b cell-wall binding domain (CBD) to anchor the enzyme onto *Staphylococcus aureus* peptidoglycan (PGN) although they are catalytically inactive. Similarly, mutagenesis in the LysGH15 SH3b domain on residues that interact with glycine pentapeptide confirmed the requirement for localization of the lysin to the cell wall (49). The structural model of SH3b2 indicates that residues predicted to be involved in interactions with the PxxP motif are not located on the same interface as the glycine pentapeptide binding interface previously described. Most peptidoglycan characterizations in the *Lactobacillus* genus indicate that they do not possess a glycine pentapeptide cross-bridge in their peptidoglycan. Instead, in species like *L. johnsonii*, the third amino acid of the peptide stem (L-lysine) is connected to the fourth amino acid (D-alanine) of an adjacent peptide stem via a single D-aspartic acid interpeptide bridge (38). This is structurally distinct from the poly-glycine cross-bridges characteristic of *Staphylococcus*. It is possible that because *L. johnsonii* lacks the poly-glycine structural substrate entirely, and because of the presence of multiple copies of SH3b domains in Sdp, these domains are under no evolutionary pressure to maintain classic extracellular PGN-binding grooves like those found in lysostaphin. This absence of local substrate binding freed the SH3b scaffold to drift and adapt to a completely new microenvironment: host-cell signaling interfaces.

The use of site-directed mutagenesis and chimera building allowed the mapping of distinct structural components that govern the SH3b2 domain mode of action. The role of the C-terminal linker sequence is significant in cellular uptake, and residues in SH3b2 are involved in host signaling. Deletion of the C-terminal linker minimized promoter activation in reporter cell lines, whereas grafting it onto the naturally inactive SH3b6 domain partially conferred signaling capabilities on that domain. Immunofluorescence assays in human THP-1 Mp visually confirmed that this C-terminal linker controls Mp entry. Once inside the cell, downstream signal transduction is driven by the hydrophobic pocket of the SH3b2 domain. Mutating (W508A) inactivated the protein, consistent with reports in eukaryotic SH3, where tryptophan residues serve as critical hydrophobic anchors that interact with host proline loops (63, 64). In addition, the replacement of the conserved G501 with a bulky, negatively charged aspartic acid (G501D) abolished the induction of the NF-κB and IRF pathways, eliminating induction of the antiviral OAS pathway and IL10. Since confocal microscopy showed that the SH3b2 carrying the G501D mutation is internalized similarly to the wild-type domain, we propose that G501 is strictly required for downstream intracellular signal transduction. However, the identity of the specific interactor host proteins as well as cellular localization during trafficking remains to be elucidated and will be the focus of future research.

Overall, this study provides a baseline for identifying and utilizing effector molecules responsible for the health benefits of probiotics. The use of these effectors can help reduce the risks associated with live probiotics or EVs in immunocompromised individuals. The potential to enhance SH3b2 activity through minor modifications underscores the need to characterize its mechanism of action. In addition, characterizing how the SH3b domain exploits molecular mimicry to safely plug into macrophage signaling networks opens a new frontier for cell-free, non-living biomimetic therapeutics.

## Data Availability

The original contributions presented in the study are included in the article/**Supplementary Material**. Further inquiries can be directed to the corresponding author.
