# Supplementary material for: Specific residues modulate the antiviral activity of the extracellular vesicle-derived effector SH3b domain from Lactobacillus johnsonii N6.2
Source: Front Immunol. 2026 Oct 22;17:1977036. doi: 10.3389/fimmu.2026.1977036 (PMC13735725; doi:10.3389/fimmu.2026.1977036)
Supplement: Supplementary file 1 [file Supplementaryfile1.docx]

Supplementary Material

130

KDa

250

180

95

72

55

43

34

26

17

10

SH3b1

SH3b2

SH3b4

SH3b3

SH3b6

SH3b5

SH3b2ΔC2

SH3b6::C2

**Supplementary Figure S1.** SDS-PAGE of purified domains of Sdp (Lane 1-6) as well SH2b6::C2 and SH3b2ΔC2 proteins (Lane 7 & 8). Proteins were added at 0.75 µg/mL.

C

**Supplementary Figure S2.** **Structural modeling of SH3b2 and SH3b6 predicts a β barrel fold in both domains.** **(A)** SH3b2 model consists of 9 β strands (labelled β1-β9) divided in 3 different β sheets where Sheet A (Magenta) is formed of β2, β5 & β9, Sheet B (Green), β3 & β4 and Sheet C (Blue) β1, β6, β7 & β8. **(B)** SH3b6 model predicts 8 β strands grouped together in 3 β sheets, where Sheet A (Blue) has β1 & β8, Sheet B (Green) β2 & β3 and Sheet C (Magenta) β5, β6 & β7. **(C)** Multiple sequence alignment (MSA) of SH3b domains showing conserved regions. The C-terminal linker is present in all the domains except for SH3b6. Structural modeling was performed using Phyre 2.2 with standard settings. Secondary structure was annotated using DSSP command in UCSF ChimeraX 1.9, which allowed the identification of β-strands and presence of loops in SH3b2 and SH3b6. MSA of SH3b domains was performed using MultAlin tool and further edited using Jalview.

**Supplementary Figure S3.** SDS-PAGE of purified mutant SH3b2 domains as indicated on top of each lane. Proteins were added at 0.75 µg/mL.

**Supplementary Figure S4.** Differential scanning fluorimetry of purified SH3b domains. Different letters above bars represent statistically significant differences at p ≤ 0.05 based on one-way ANOVA followed by Tukey’s *post-hoc* test.

B

A

**Supplementary Figure S5.** **Mp cell viability was maintained after SH3b2 or mutant variants exposure for 18 h.** Resazurin reduction assay was performed using 1.5 (**A**) and 5 µg/mL (**B**) of purified proteins. THP-1 Mp were treated with proteins for 18 h at 37 ˚C and 5% CO_2._ Next, old media was replaced with new media containing 15 µg/mL of resazurin reduction salt solution (from 0.2 mg/mL stock prepared in PBS). Fluorescence was measured 4 h post incubation at 37 ˚C and 5% CO_2_ using Agilent BioTek Synergy H1 plate reader with default resazurin fluorophore settings (Agilent, CA, USA). Different letters above bars represent statistically significant differences at p ≤ 0.05 based on one-way ANOVA followed by Tukey’s *post-hoc* test.

**Supplementary Figure S6. Stitched merged images obtained from confocal microscopy.** THP-1 Mp cells were treated with 1.5 µg/mL of proteins for 1 h at 37 ˚C and 5% CO_2_ or kept untreated as a control. Slides were prepared as indicated in the methodology section. Stitched images were created using Leica LAS X Navigator in Stellaris mode.

**Supplementary Figure S7.** **Representative images via orthogonal sectioning of Z-stacks depicts the role of C-terminal linker of SH3b2 in protein uptake.** Representative confocal microscopy Z-stack orthogonal sections were obtained using recombinant protein fusions to the C-terminal of mCherry (mCh). Mp cells were treated with 5 µg/mL of either purified mCh or mCh-SH3b2, mCh-SH3b6, mCh-SH3b2ΔC2, mCh-SH3b6::C2 for 1 h, followed by slide preparation as discussed in the methodology section. Staining was performed for THP-1 nuclei (DAPI, Blue), THP-1 cell membrane (Alexa488, Green, WGA), mCherry fusion proteins (Red). Z-step size was set between 0.2-0.3 with 25-35 steps for all images. Orthogonal sections were captured using Leica LAS X (version 4x) Orthogonal Sections tool for Z-stacks.
